# Celecoxib inhibits growth of human autosomal dominant polycystic kidney cyst-lining epithelial cells through the VEGF/Raf/MAPK/ERK signaling pathway

**DOI:** 10.1007/s11033-012-1611-2

**Published:** 2012-03-14

**Authors:** Tao Xu, Nian-Song Wang, Li-Li Fu, Chao-Yang Ye, Sheng-Qiang Yu, Chang-Lin Mei

**Affiliations:** 1 Department of Nephrology, Shanghai Jiao Tong University Affiliated Sixth People’s Hospital, Shanghai, 200233 China; 2 Division of Nephrology, Changzheng Hospital, Second Military Medical University, Shanghai, 200003 China

**Keywords:** Celecoxib, COX-2, Autosomal dominant polycystic kidney disease, Proliferation, Apoptosis

## Abstract

Autosomal dominant polycystic kidney disease (ADPKD) is a progressive chronic kidney disease. To date there are no effective medicines to halt development and growth of cysts. In the present study, we explored novel effects of celecoxib (CXB), a COX-2 specific inhibitor, on primary cultures of human ADPKD cyst-lining epithelial cells. Primary cultures of ADPKD cyst-lining epithelial cells were obtained from five patients. Effects of CXB were measured by various assays to detect BrdU incorporation, apoptosis and proliferation in vitro. Additionally, effects of CXB on kidney weight, the cyst index, the fibrosis index, blood urea nitrogen (BUN), serum creatinine (SCr), serum 6-keto-PGF-1α, serum thromboxane-2 (TXB2) and renal PCNA expression were assessed in Han:SPRD rat, a well-characterized rodent model of PKD. CXB inhibited proliferation of ADPKD cyst-lining epithelial cells, blocked the release of VEGF from the cells and induced extensive apoptosis in a time- and dose-dependent manner. Moreover, CXB up-regulated the cell cycle negative regulator p21^CIP/WAF1^ and the cell cycle positive regulator Cyclin A, blocked ERK1/2 phosphorylation, induced apoptotic factors (Bax and caspase-3) and reduced Bcl-2. Furthermore, CXB inhibited the expression of VEGFR-2 and Raf-1 in ADPKD cyst-lining epithelial cells. CXB markedly reduced the cyst index, the fibrosis index, leukocyte infiltration, BUN, SCr, serum 6-keto-PGF-1α, TXB2 and renal PCNA expression in Han:SPRD rat. We demonstrated for the first time that CXB could suppress renal cyst-lining growth both in vitro and in vivo in Han:SPRD rat. CXB can inhibit proliferation, suppress cell cycle progression, and induce apoptosis in ADPKD cyst-lining epithelial cells through the inhibition of the VEGF/VEGFR-2/Raf-1/MAPK/ERK signaling pathway.

## Introduction

Celecoxib (CXB), a highly selective cyclooxygenase (COX-2) inhibitor, is a sulfa non-steroidal anti-inflammatory drug (NSAID) used in the treatment of rheumatoid arthritis, osteoarthritis, acute pain [1], amyotrophic lateral sclerosis [2], and painful menstruation and menstrual symptoms. Furthermore, it reduces the number of colon and rectum polyps in patients with familial adenomatous polyposis [3]. CXB was reported to significantly inhibit the growth of colon or rectal cancer tumor cells [4, 5], head and neck cancer cells [6], leukemia cells [7], lung cancer cells [8] and others. In fact, CXB has recently been clinically evaluated for its antitumor effects [9, 10]. In the kidney, a significant association between maximal COX-2 immunostaining and clinical response was observed in renal cell carcinoma [11]. However, continuous therapy with low-dose cyclophosphamide and CXB was reported to have limited efficacy in treating renal cell carcinoma [12].

Autosomal dominant polycystic kidney disease (ADPKD) is the most common kidney hereditary disorder that occurs in 1:1,000 individuals and is responsible for 10% of end-stage renal disease (ESRD) [13]. Its symptoms may vary from none to a variety of problems such as bleeding, pain, urinary infection, kidney stones and kidney failure [14–16]. Genetically, mutations in the gene on chromosome 16, which codes for the protein polycystin 1, or the *PKD2* gene on chromosome 4, which codes for the protein polycystin 2, cause ADPKD. Only a few familial cases are unrelated to either locus [17]. To date, effective clinical interventions are minimal [18]. It was reported that COX-2 had high expression in the kidney of an ADPKD animal model [19], suggesting that COX-2 might be implicated in the pathophysiology of ADPKD, thereby making COX-2 an attractive therapeutic target. In fact, it was recently reported that NS-398, a selective COX-2 inhibitor, markedly slowed disease progression and attenuated altered prostanoid production in a rat model [20]. However, the underlying mechanism remains unclear. More importantly, the effects of COX-2 in human ADPKD cells are unknown. In this study, we report that inhibition of COX-2 with CXB prevents growth of human ADPKD cyst-lining epithelial cells by targeting cell cycle and apoptotic pathways, which may provide new insight for future treatment of ADPKD.

## Materials and methods

### Patients and cell culture

ADPKD cyst-lining epithelial tissues were obtained from five individuals with histologically confirmed ADPKD. Cells were isolated and cultured as described previously [21]. Ethical committee approval for tissue collection was obtained from the Second Military Medical University Affiliated Changzheng Hospital. Informed consent was obtained from every patient. After nephrectomy, the lesion tissues were isolated, cut into pieces, and then digested with collagenase (0.1%) in a 37°C incubator for 1 h. ADPKD cyst-lining epithelial cells were incubated in a CO_2_ incubator (37°C; 5% CO_2_ in air) and cultured in Dulbecco’s modified eagle’s medium (DMEM) plus 10% fetal bovine serum (FBS). The third passage of cultured cells in the logarithmic growth phase was randomly divided into groups for further treatments.

### BrdU cell proliferation assay

A BrdU cell proliferation assay was performed as reported previously [22]. Cells were seeded into 96-well plates at a density of 3 × 10^4^ cells/well. When the culture reached 80% confluence, serum-free DMEM + F12 (GIBCO, USA) medium was added. After synchronization for 24 h, CXB (Pfizer, USA) at various concentrations (0, 2.5 × 10^−6^, 5 × 10^−6^, 1 × 10^−5^, 2 × 10^−5^, 3 × 10^−5^, 4 × 10^−5^ and 5 × 10^−5^ mol/l) was added to the media. Cells continued to grow for 24, 48 and 72 h. BrdU-labeled working solution (10 μl; Roche, Switzerland) was then added to each well according to the manufacturer’s protocol. Absorbance was measured at 405 nm to determine the BrdU concentration in each sample. An absorbance measurement at 490 nm served as a control.

### Assessment of vascular endothelial growth factor (VEGF) and PGE_2_ secretion

Human polycystic kidney cyst-lining epithelial cells were seeded into three 6-well plates at a density of 5 × 10^4^ cells/well. For each plate, six different concentrations of CXB (0, 1 × 10^−6^, 5 × 10^−6^, 1 × 10^−5^, 2 × 10^−5^ and 3 × 10^−5^ mol/l) were added. Three plates were cultured for 12, 24 and 48 h. The supernatant of each well was collected to detect VEGF and PGE_2_ absorbance values (A) using an enzyme-linked immunosorbent assay (ELISA) kit (Roche, Switzerland) at 450 nm [23].

### Assessment of cell cycle and proliferation

Human polycystic kidney cyst-lining epithelial cells were seeded into three 6-well plates at a density of 4 × 10^4^ cells/well. CXB was added at three different concentrations (0, 1 × 10^−5^, 2 × 10^−5^ mol/l). Plates were incubated at 37°C for 24, 48 and 72 h. Cells were then harvested and washed in phosphate buffered saline (PBS) followed by ethanol fixation. Cells were stained with propidium iodide (PI) before flow cytometry (Becton–Dickinson) analysis as reported previously [24].

### RNA isolation and qPCR analysis

Human polycystic kidney cyst-lining epithelial cells were seeded into three 60-mm dishes at a density of 5 × 10^4^ cells/dish. CXB was added to the dishes at three different concentrations (0, 1 × 10^−5^, 2 × 10^−5^ mol/l). Cells were incubated for 24 h and then washed three times in cooled PBS. Total RNA was extracted with Trizol (Takara, Japan). The quality of each RNA sample was assessed by electrophoresis. RNA was reverse transcribed to complementary DNA (cDNA) as previously reported [25]. cDNA was synthesized in 20 μl of reaction mixture from 2 μg of RNA. Quantitative (or real-time, q) PCR (Applied Biosystems) was completed using SYBR Green I (BioRad, USA). The housekeeping gene GAPDH was used as an internal control [26] to estimate relative quantity of mRNA expression and correct for differences in sample volumes Table 1.

### Apoptosis assays

Apoptosis in ADPKD cells was evaluated by terminal deoxynucleotide transferase-mediated dUTP nick end-labeling (TUNEL) staining and Annexin V + PI staining analysis. TUNEL was used to detect the cellular apoptosis rate as reported previously [27]. Procedures were carried out according to the kit instructions (Roche, Switzerland). TUNEL reaction solution was substituted with TdT-free solution for a negative control. Sections were pretreated 10 min with DNase and visualized by diaminobenzidine (DAB) staining. Positive nuclei were identified by brown color. The calculation method for determining the number of apoptotic cells was as follows: 10 fields of vision under microscopy (×400) were randomly selected and the apoptosis rate (%) = (the number of positive cells/the total number of ADPKD cyst-lining epithelial cells) × 100%. For Annexin V/PI staining analysis, the Alexa Fluor 488 labeled-Annexin V and PI assay (Invitrogen, Carlsbad, CA, USA) were used according to the manufacturer’s instruction. A BD FACScalibur flow cytometer was used.

### Western blot analysis

Western blot analysis was performed as described previously [28, 29]. Human polycystic kidney cyst-lining epithelial cells were seeded into five 60 mm culture dishes at a density of 5 × 10^4^ cells/dish. CXB (2 × 10^−5^ mol/l) was added to each dish and incubated for 0, 12, 24, 36 and 48 h. In an additional experiment, cells were stimulated by VEGF (40 ng/ml) for 24 h and then treated with CXB (20 μM) and a Raf/VEGFR2 inhibitor (NVP-AAL881, 2 μM) for 48 h. Cells were then lysed and total protein was extracted. Membrane protein was extracted using the Membrane and Cytoplasmic Protein Extraction Kit (Sangon, China, catalogue: BSP005). Protein concentration was measured with a BCA protein assay kit (Pierce, USA). Protein (40 μg) from each dish was loaded for polyacrylamide gel electrophoresis (SDS-PAGE, resolving gel concentration was 12%) and transferred to nitrocellulose membranes at 100 V for 2 h. The membrane was blocked by treatment with 5% nonfat milk for 1 h at room temperature and was probed at 4°C overnight with 11 different primary antibodies: mouse anti-human PCNA (1:200, Santa Cruz), mouse anti-human ERK1/2 (1:2,000, Cell Signaling Technology), rabbit anti-human phospho-ERK1/2 (1:1,000, Cell Signaling Technology), mouse anti-human p21^CIP/WAF1^ (1:200, Santa Cruz), mouse anti-human Cyclin A (1:1,000), mouse anti-human Bax (1:100, Santa Cruz), mouse anti-human Bcl-2 (1:100, Santa Cruz), mouse anti-human caspase-3 (1:800, Santa Cruz), rabbit anti-human VEGFR-2 (1:800, Santa Cruz), rabbit anti-human Raf-1 (1:800, Santa Cruz) and mouse anti-human GAPDH (Sigma). After washing with PBS, the membrane was incubated with the corresponding HRP-labeled secondary antibody for 2 h at room temperature. The membrane was washed in PBS again and then transferred to ECL working solution (Pierce). The film was exposed and scanned. The optical density of protein bands was quantified and GAPDH protein served as the internal control [30].

### In vivo study in heterozygous (Cy/+) Han:SPRD rat

The (Cy/+) Han:SPRD rat is a well-established animal model for PKD. Three-week-old (Cy/+) male rats were randomly divided into three groups: a control group, a low-dose CXB (30 mg/kg/day) group and a high-dose CXB (100 mg/kg/day) group. The control group received a normal diet while the latter two groups received a normal diet plus CXB. Blood was obtained from the vena orbitalis posterior at 4, 6, 8, 10, 12 and 16 weeks of treatment. Blood urea nitrogen (BUN) and serum creatinine (SCr) were determined with an automatic biochemistry analyzer (Hitach 7080, Tokyo, Japan). Serum 6-keto-PGF-1α and serum thromboxane-2 (TXB2) were measured using commercial ELISA kits (Cayman,USA). After 16 weeks, the rats were anesthetized with sodium pentobarbital (40 mg/kg) and the kidneys were removed. Fresh kidneys were isolated and cut into three parts. One portion of the tissues was fixed in 10% formalin and subjected to hematoxylin–eosin (HE) staining, periodic acid-Schiff (PAS) staining and Masson staining as described previously [31] to evaluate the cyst index, the fibrosis index and leukocyte infiltration. A second portion of the tissues was used to perform immunohistochemistry as described previously [32]. The last portion of the tissues was homogenized and then subjected to immunoblot analysis of phospho-ERK and Raf-1 expression.

### Statistical analysis

All data were expressed as means ± SD. Paired comparison was performed using one-way analysis of variance (ANOVA) or two-way ANOVA followed by the SNK *t* test (SPSS version 11, Chicago, IL, USA). *P* < 0.05 was considered statistically significant.

## Results

### CXB inhibits ADPDK cell proliferation

**Fig. 1:**
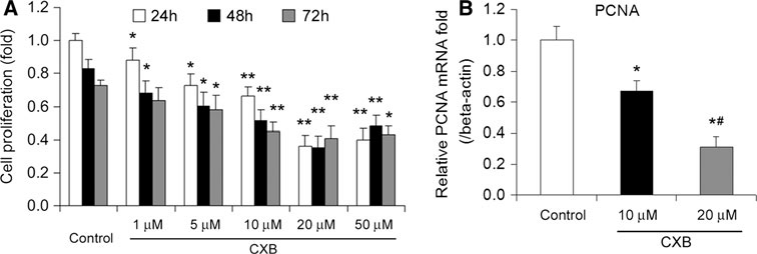
Cell proliferation in ADPKD cells. **a** ADPKD cyst-lining epithelial cell proliferation was determined by a BrdU assay. **b** PCNA mRNA expression was determined using real-time PCR. *P < 0.05, **P < 0.01 vs. control; *n* = 8

As shown in Fig. 1a, different concentrations of CXB ranging from 1 to 50 μM significantly inhibited ADPKD cell proliferation in a dose-dependent manner. No significant differences were observed between responses to 20 and 50 μM of CXB, suggesting 20 μM might be the maximum effective dose of CXB in ADPDK cells. Moreover, we evaluated the effect of CXB on PCNA mRNA expression, a marker for cell proliferation (Fig. 1b). PCNA expression was significantly reduced in ADPKD cyst-lining epithelial cells when treated with 10 μM CXB, and was further decreased when treated with 20 μM CXB.

### CXB inhibits VEGF and PGE_2_ secretion

**Fig. 2:**
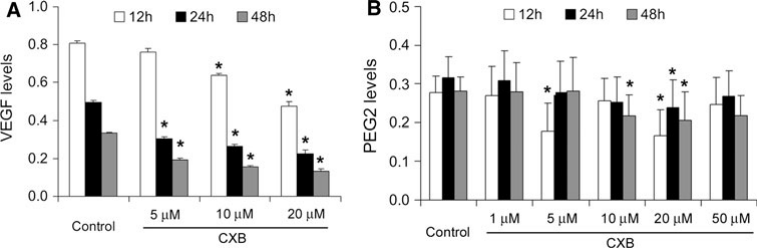
Effects of CXB on secretion of VEGF and PGE_2_ from ADPKD cells. Levels of VEGF (**a**) and PGE_2_ (**b**) in media were determined by ELISA. *P < 0.05, **P < 0.01 vs. control; *n* = 8

CXB significantly inhibited VEGF release from cultured ADPKD cyst-lining epithelial cells. As shown in Fig. 2a, 20 μM CXB resulted in a maximum inhibitory effect at 48 h (*P* < 0.05). The inhibitory effect occurred in a time- and dose-dependent manner. In contrast, the inhibitory effect of CXB on PGE_2_ secretion was neither time- nor dose-dependent (Fig. 2b). CXB at 20 μM reached a maximum inhibitory effect at 12 h (*P* < 0.05).

### CXB induces cell cycle arrest

**Fig. 3:**
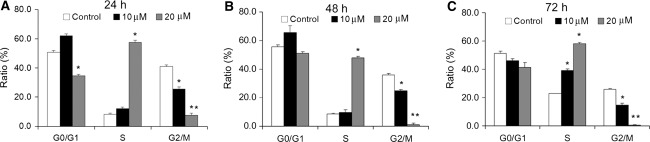
Effects of CXB on cell cycle distribution of ADPKD cells. Cell cycle status of ADPKD cells was determined using PI staining and flow cytometry. Cells were treated with CXB for 24 h (**a**), 48 h (**b**) and 72 h (**c**). *P < 0.05, **P < 0.01 vs. control; *n* = 8

As shown in Fig. 3, a 20 μM CXB treatment for 24 h induced cell cycle arrest at the S phase (Fig. 3a, *P* < 0.05), while the number of G2/M phase cells decreased (Fig. 3a, *P* < 0.05). This effect on cell cycle was less in cells incubated with 10 μM CXB (Fig. 3a). When the concentration of CXB and exposure time increased, the effects of cell cycle arrest became more apparent (48 h, Fig. 3b; 72 h, Fig. 3c).

### CXB treatment induces apoptosis in ADPKD cyst-lining epithelial cells

**Fig. 4:**
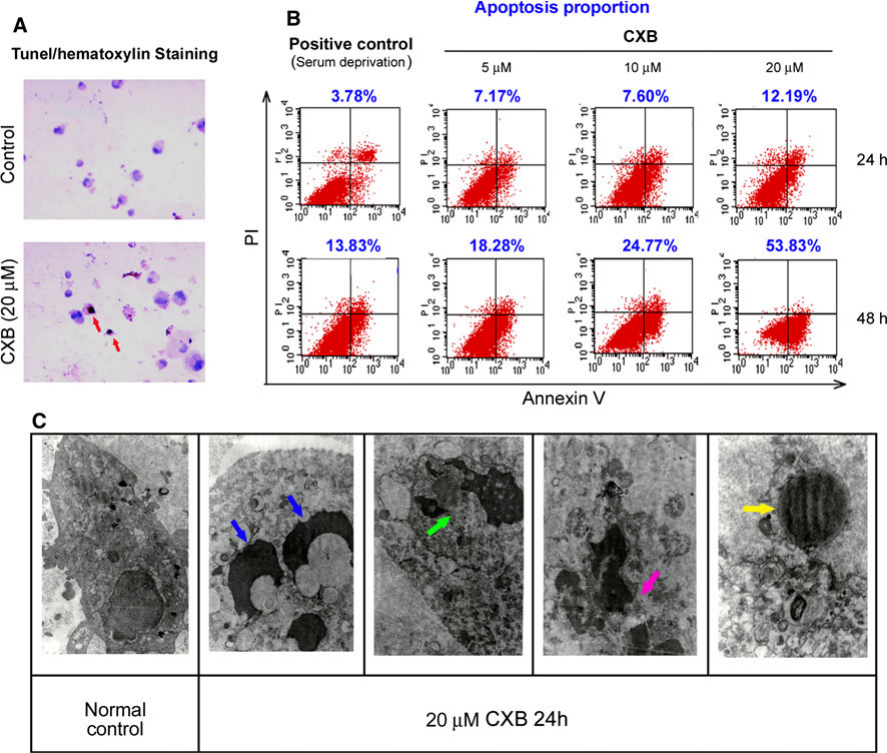
Induction of apoptosis in ADPKD cells by CXB. Apoptosis assays showing CXB induces ADPKD cell apoptosis. **a** Apoptosis was evaluated by TUNEL staining. The brown nuclear area indicates TUNEL-positive cells (*red arrows*). **b** Apoptosis was evaluated by Annexin V + PI staining with a flow cytometer. **c** Apoptosis was evaluated by electron microscopy. (Color figure online)

As shown in Fig. 4a, there were significantly more cells stained with DAB in apoptotic ADPKD cyst-lining epithelial cells (red arrow, 28.5 ± 1.6% vs. 2.8 ± 0.2%, *P* < 0.01) than in control cells. Under the microscope, typical apoptotic phenomena (brown nucleus, nuclear condensation, chromatin margination, condensation and scattered brown apoptotic bodies) were identified in CXB-treated cells. Apoptotic rates significantly differed between treatment groups. As shown in Fig. 4b, Annexin V + PI staining showed that CXB (5, 10 and 20 μM) induced marked increases in the number of apoptotic cells in a time- and dose-dependent manner. Increases in apoptotic cells were also noted with serum starvation (positive control). Incubation with 20 μM CXB for 48 h induced maximal apoptosis (53.8%). Chromatin aggregation (blue arrows), apoptotic bodies (green arrow) and naked nuclei (yellow arrow) were observed by electron microscopy in cells treated with 20 μM CXB for 24 h (Fig. 4c).

### Effects of CXB on mRNA expression of caspase-3, Bax and Bcl-2

**Fig. 5:**
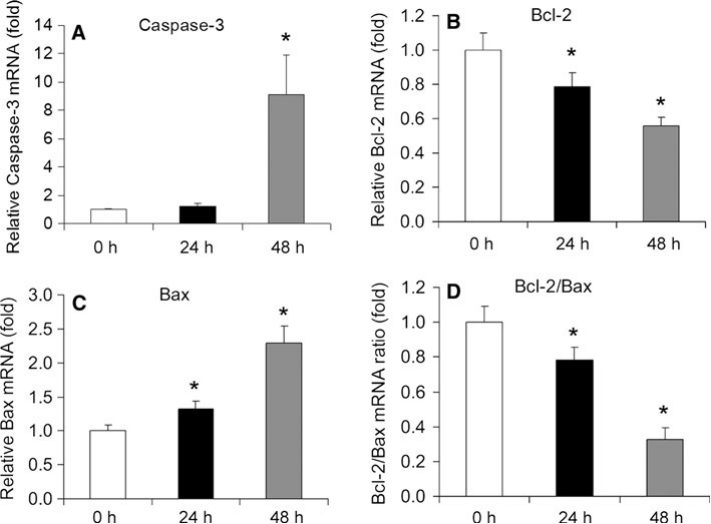
Gene expression of apoptosis regulatory proteins. Effects of CXB (20 μM) on mRNA expression of caspase-3 (**a**), Bax (**b**), Bcl-2 (**c**), and the Bcl-2/Bax ratio (**d**). *P < 0.05 vs. control. *n* = 8. GAPDH was used as a loading control

After 24 h of CXB (20 μM) treatment, caspase-3 mRNA expression did not change (Fig. 5a). However, there was significant up-regulation of the apoptotic gene Bax (Fig. 5c) and down-regulation of the anti-apoptotic gene Bcl-2 (Fig. 5b), which resulted in a decrease in the Bcl-2/Bax ratio (Fig. 5d, *P* < 0.05). After 48 h of treatment, the mRNA level of caspase-3 was markedly increased (Fig. 5a) while the changes in Bax and Bcl-2 remained the same as at the earlier time point.

### Effects of CXB on protein expression of ERK1/2 signaling pathway and other apoptosis-related proteins

**Fig. 6:**
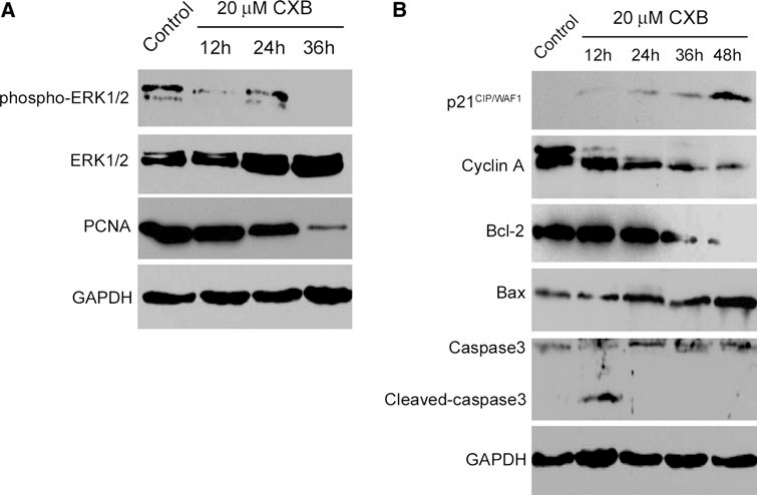
Protein expression of cell cycle and apoptosis regulatory proteins. Effects of CXB (20 μM) on ERK1/2 signaling pathway phosphorylation (**a**), PCNA expression (**a**), Bax/Bcl-2 expression (**b**), caspase-3 cleavage (**b**), Cyclin A expression (**b**) and p21^CIP/WAF1^ expression (**b**). GAPDH was used as a loading control. *P < 0.05 vs. control; *n* = 8. GAPDH served as a loading control

As shown in Fig. 6a, CXB (20 μM) suppressed the phosphorylation of ERK1/2 in a time-dependent manner as compared to that in the serum-free control group, while total ERK1/2 protein exhibited no significant differences between treatment groups or across treatment times. Similarly, PCNA protein expression was also decreased (Table 1). As shown in Fig. 6b, CXB increased the expression of p21^CIP/WAF1^ and Bax, whereas CXB decreased the expression of Cyclin A and Bcl-2 in a time-dependent manner. Total caspase-3 (precursor, 34 kDa) expression was enhanced after 24 h of treatment, while the expression of cleaved caspase-3 (17 kDa) was observed only at the 12 h time point.

**Table 1:** Primer sets for qRT-PCR

Primer name	Sequence	Length (bp)
***PCNA***	F: 5′-TCATTACACTAAGGGCCGAAG-3′	170
	R: 5′-TTCACCAGAAGGCATCTTTACT-3′	
***Bax***	F: 5′-GCAAACTGGTGCTCAAGG-3′	192
	R: 5′-CGCCACAAAGATGGTCAC-3′	
***Bcl-2***	F: 5′-GCCTTCTTTGAGTTCGGTG-3′	207
	R: 5′-CAGAGACAGCCAGGAGAAATC-3′	
***Caspase 3***	F: 5′-CAGAACTGGACTGTGGCATTG-3′	192
	R: 5′-GCTTGTCGGCATACTGTTTCA-3′	
***GAPDH***	F: 5′-GGT ATC GTG GAA GGA CTC ATG AC-3′	188
	R: 5′-ATG CCA GTG AGC TTC CCG TTC AGC-3′	

### Effects of CXB on VEGF-related signaling pathway in vitro

**Fig. 7:**
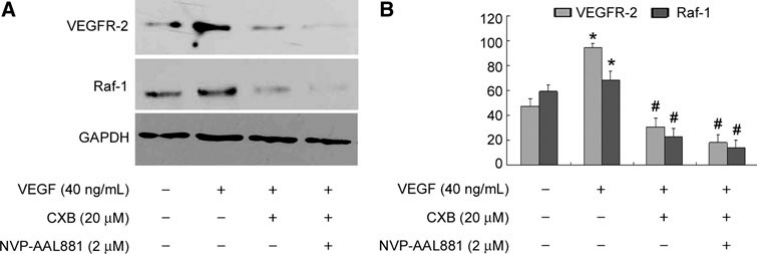
Effects of CXB (20 μM) on VEGF-related signaling proteins under VEGF stimulation in vitro. Cells were pre-treated with VEGF (40 ng/ml) for 24 h and then incubated with CXB (20 μM) or NVP-AAL881 (2 μM) for 48 h. **a** Representative images of immunoblotting of VEGFR-2 and Raf-1. **b** Quantitative analysis of protein expression of VEGFR-2 and Raf-1. *P < 0.05 vs. control; ^#^ *P* < 0.05 vs. VEGF stimulation; *n* = 8. GAPDH served as a loading control

As shown in Fig. 7a, b, VEGF treatment stimulated VEGFR-2 and Raf-1 expression. Both CXB (20 μM) and the VEGFR-2/Raf-1 signaling pathway inhibitor NVP-AAL881 suppressed the up-regulation of VEGFR-2 and Raf-1.

### Effects of CXB on VEGF-Raf-1-ERK1/2 signaling pathway in (Cy/+) Han:SPRD rat

**Fig. 8:**
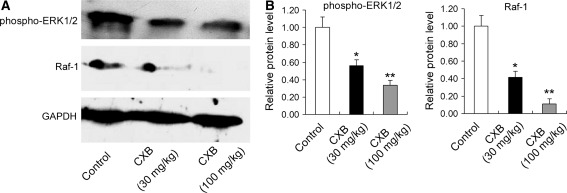
Protein expression of phospho-ERK1/2 and Raf-1 in vivo. Effects of low-dose (30 mg/kg) and high-dose (100 mg/kg) CXB on phospho-ERK1/2 and Raf-1 in heterozygous (Cy/+) Han:SPRD rat. **a** Representative images of immunoblotting of phospho-ERK1/2 and Raf-1. **b** Quantitative analysis of protein expression of phospho-ERK1/2 and Raf-1. *P < 0.05 vs. control; **P < 0.01 vs. control; *n* = 6 per group. GAPDH served as a loading control

As shown in Fig. 8a, b, diets supplemented with either of the two doses of CXB (30 and 100 mg/kg) suppressed the expression of phospho-ERK1/2 and Raf-1 in Han:SPRD rat.

### Effects of CXB on kidney weight and serum parameters in Han:SPRD rat

**Fig. 9:**
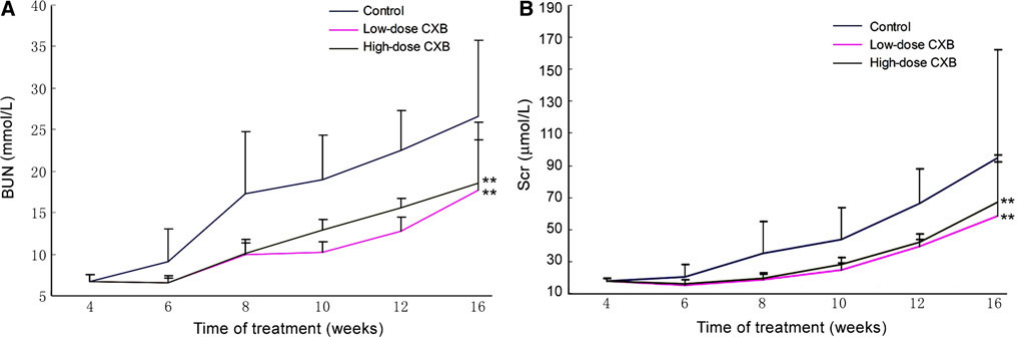
Effects of CXB on BUN and SCr levels in Han:SPRD rats. Effects of low-dose (30 mg/kg) and high-dose (100 mg/kg) CXB on BUN (**a**) and SCr (**b**) levels in Han:SPRD rat. **P < 0.01 vs. control; *n* = 6 per group

As shown in Table 2, diets supplemented with either of the two doses of CXB (30 and 100 mg/kg) attenuated the increase in kidney weight in Han:SPRD rat. Moreover, both doses of CXB (30 and 100 mg/kg) significantly decreased the levels of serum 6-keto-PGF-1α and serum TXB2 in Han:SPRD rat (Table 3). We also measured the BUN and SCr levels. As shown in Fig. 9, the BUN and SCr levels steadily increased in Han:SPRD rat, and these effects were attenuated by both doses of CXB.

**Table 2:** Characteristics of (Cy/+) Han:SPRD rat after 16 weeks of treatment

Group	Body weight (g)	Right kidney weight (g)	Left kidney weight (g)	Kidney weight/body weight (%)
Control	434 ± 33	2.89 ± 0.91	2.87 ± 0.71	1.33 ± 0.48
Low-dose CXB	416 ± 18	2.26 ± 0.53*	2.34 ± 0.34*	1.11 ± 0.49*
High-dose CXB	401 ± 32*	2.28 ± 0.25*	2.33 ± 0.54*	1.14 ± 0.25*

* *P* < 0.05 vs. control

**Table 3:** Characteristics of (Cy/+) Han:SPRD rat after 16 weeks of treatment

Group	Serum 6-keto-PGF-1α (pg/ml)		Serum TXB2 (pg/ml)	
	12 weeks	16 weeks	12 weeks	16 weeks
Control	1830 ± 807	2790 ± 830	139 ± 14	249 ± 94
Low-dose CXB	1380 ± 758*	1830 ± 233*	128 ± 6*	157 ± 9**
High-dose CXB	1310 ± 455*	1150 ± 106*	125 ± 7*	158 ± 10**

*P < 0.05 vs. control, **P < 0.01

### Effects of CXB on kidney morphology and expression of PCNA in Han:SPRD rat

**Fig. 10:**
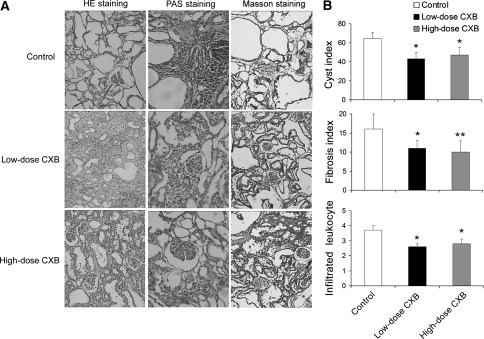
**a** Representative images of HE, PAS and Masson staining of kidneys from Han:SPRD rat treated with vehicle, low-dose (30 mg/kg) and high-dose (100 mg/kg) CXB. **b** Quantitative analysis of cyst index, fibrosis index and leukocyte infiltration. *P < 0.05 vs. control; **P < 0.01 vs. control; *n* = 6 per group

**Fig. 11:**
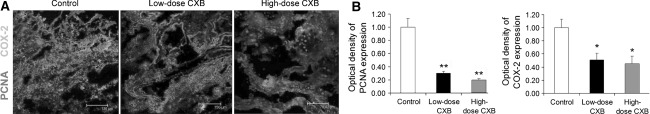
**a** Representative images of immunohistochemistry of PCNA (*red*) and COX-2 (*green*) in Han:SPRD rat treated with vehicle, low-dose (30 mg/kg) and high-dose (100 mg/kg) CXB. **b** Quantitative analysis of PCNA and COX-2 expression in Han:SPRD rat treated with vehicle, low-dose (30 mg/kg) and high-dose (100 mg/kg) CXB. *P < 0.05 vs. control; **P < 0.01 vs. control; *n* = 6 per group. (Color figure online)

We applied three staining methods (HE, PAS and Masson staining, Fig. 10a) to study kidney morphology and calculate the cyst index, the fibrosis index and leukocyte infiltration. Both doses of CXB successfully decreased the cyst index, the fibrosis index and leukocyte infiltration (Fig. 10b). Finally, CXB markedly inhibited expression of PCNA (a marker of cellular proliferation) and COX-2 in (Cy/+) Han:SPRD rat (Fig. 11a, b).

## Discussion

In the present study, we showed that CXB suppressed the proliferation of human ADPKD cyst-lining epithelial cells by targeting cell cycle and apoptosis pathways in a time- and dose-dependant manner. This is the first report suggesting an anti-growth effect of CXB in human ADPKD cyst-lining epithelial cells. Although it is the fourth leading cause of ESRD, there are still no effective therapeutic interventions for treatment of ADPKD, with the exception of gene replacement therapy. Thus, we provide additional evidence for the application of COX-2 inhibitors in the treatment for ADPKD.

Raz et al*.* [33] speculated that COX-2 inhibitors functioned as anti-cancer agents primarily through two mechanisms: a COX-dependent pathway by inhibiting the COX enzyme (primarily COX-2), and a COX-2 independent pathway that induces blockage of cell growth at different stages of reproduction and causes apoptosis. According to our results, growth inhibition of human ADPKD cyst-lining epithelial cells induced by CXB may be due to its pro-apoptotic and cell cycle arrest properties. Several previous reports have indicated that CXB can induce apoptosis in various tumor cells [34, 35]. Using the TUNEL method, Annexin V + PI staining and electron microscopy, we observe that CXB indeed induces apoptosis of ADPKD cyst-lining epithelial cells.

Moreover, we studied the signals underlying the pro-apoptotic ability of CXB. We found that CXB could inhibit the release of VEGF from ADPKD cyst-lining epithelial cells in a time- and dose-dependant manner. Additionally, CXB also inhibited secretion of PGE_2_, but in a time- and dose-independent manner. It is well known that VEGF is important for the regeneration of ADPKD cyst-lining epithelial cells, the secretion of cystic fluid, hyperplasia of extracellular matrix, and the growth of new blood vessels [36]. VEGF can bind to VEGFR-2 and activate Ras, thereby further activating Raf-1. Raf-1 can then stimulate MAPK signaling cascades [37]. In pancreatic cancer, Ras/Raf-1/MAPK signaling is required for mediating growth factor-induced (such as epidermal growth factor) oncogenic effects. Activation of this pathway is associated with tumor progression (proliferation) and invasiveness (cell migration) [38–40].

We found that VEGF secreted by cultured ADPKD cyst-lining epithelial cells bound to VEGFR-2 and directly activated the Ras/Raf-1/MAPK pathway to induce abnormal proliferation. CXB successfully blocked this cascade and inhibited proliferation of ADPKD cyst-lining epithelial cells both in vitro and in vivo in Han:SPRD rat. There are three parallel MAPK signaling cascades, the ERK, JNK and p38 MAPK pathways. It is well known that inactivation of both the JNK and ERK pathway inhibits cell proliferation, while the ERK and p38 MAPK pathways both regulate the cell cycle. ERK1/2 is an important regulator of apoptosis in tumor cells and Bcl-2/Bax is a downstream component in the ERK1/2 signal transduction pathway [41]. Under normal circumstances, caspase-3 is an inactive pro-enzyme in the cytoplasm. Only when apoptosis is activated is caspase-3 transformed to the active form [42]. In the CXB treatment group, the ratio of Bcl-2/Bax and the amount of phospho-ERK1/2 were decreased in time-dependent manners, suggesting that CXB might increased the sensitivity of ADPKD cyst-lining epithelial cells to apoptotic stimuli. However, regarding whether CXB coordinates the three downstream pathways to inhibit cell proliferation, induce apoptosis and regulate cell cycle remains unclear and warrants further investigation.

CXB-induced cell cycle arrest, in particular the marked increase in S phase arrest in ADPKD cells, was an important finding in this study. This suggests that the ADPKD cyst-lining epithelial cells were blocked before G2/M phase, resulting in an accumulation of cells at the S phase. The proportion of cells in the S phase indicates the proportion of cells in a proliferative stage because cycle status is not only directly related to cell proliferation, but also to cell differentiation and apoptosis. The G1/S check point is crucial for cell proliferation as it controls the passage of eukaryotic cells from the first ‘gap’ phase (G1) into the DNA synthesis phase (S), which involves interaction between the positive regulator CDK2-Cyclin A (also as a G1/S checkpoint regulator) and the negative regulator p21^CIP/WAF1^ [37]. In the present study, protein expression of p21^CIP/WAF1^ in the ADPKD cells was increased whereas that of the positive regulator Cyclin A was decreased after CXB treatment, further indicating that CXB might have inhibited the proliferation of ADPKD cells by targeting the cell cycle.

Finally, we demonstrated the efficacy of CXB in (Cy/+) Han:SPRD rat, a frequently used animal model of PKD. CXB remarkably decreased the increases in kidney weight, serum 6-keto-PGF-1α, serum TXB2, BUN, SCr, the cyst index, the fibrosis index, leukocyte infiltration and PCNA expression. All these in vivo data clearly showed the potent and critically beneficial effects of CXB on ADPKD. Notably, everolimus decreased kidney growth whereas renal function declined in human ADPKD, suggesting that possible therapeutic compounds can cause kidney injury and halt kidney growth. Whether CXB can induce renal injury needs further investigation in the future.

In summary, our in vitro and in vivo findings provide the first direct evidence that CXB suppresses renal cyst-lining growth in ADPKD. Potential mechanisms of action may include induction of cell cycle arrest and apoptosis, which are achieved by the blockade of binding between VEGF and VEGFR-2, and subsequent Raf-1/ERK signaling.
